# Predator mediated selection and the impact of developmental stage on viability in wood frog tadpoles (*Rana sylvatica*)

**DOI:** 10.1186/1471-2148-11-353

**Published:** 2011-12-07

**Authors:** Ryan Calsbeek, Shawn Kuchta

**Affiliations:** 1 Department of Biological Sciences, Dartmouth College, Hanover, NH 03755 USA; 2 National Evolutionary Synthesis Center, Durham, NC 27606 USA; 3 Department of Biological Sciences, Ohio University, Athens, OH 45701 USA

## Abstract

**Background:**

Complex life histories require adaptation of a single organism for multiple ecological niches. Transitions between life stages, however, may expose individuals to an increased risk of mortality, as the process of metamorphosis typically includes developmental stages that function relatively poorly in both the pre- and post-metamorphic habitat. We studied predator-mediated selection on tadpoles of the wood frog, *Rana sylvatica*, to identify this hypothesized period of differential predation risk and estimate its ontogenetic onset. We reared tadpoles in replicated mesocosms in the presence of the larval odonate *Anax junius*, a known tadpole predator.

**Results:**

The probability of tadpole survival increased with increasing age and size, but declined steeply at the point in development where hind limbs began to erupt from the body wall. Selection gradient analyses indicate that natural selection favored tadpoles with short, deep tail fins. Tadpoles resorb their tails as they progress toward metamorphosis, which may have led to the observed decrease in survivorship. Path models revealed that selection acted directly on tail morphology, rather than through its indirect influence on swimming performance.

**Conclusions:**

This is consistent with the hypothesis that tail morphology influences predation rates by reducing the probability a predator strikes the head or body.

## Background

Many organisms exploit different environments over the course of their life cycle. Perhaps the most extreme example of this shift in resource use is that which accompanies metamorphosis in animals with complex life cycles [1]. Complex life cycles - hereafter referring to organisms with at least two discrete post-embryonic life-stages [2,3] - are ubiquitous in animals, being expressed in at least 80% of all species [4,5]. They may evolve for several reasons, such as trophic switching or specialized dispersal/breeding forms [6]. The tradeoffs that accompany shifts in niche occupancy will typically be accompanied by divergent selective regimes and alternative adaptations. In part, this accounts for the large differences in morphology, physiology, behavior, and other aspects of the phenotype observed among life stages. Although dramatically divergent morphologies among different life stages allow individuals to exploit multiple kinds of resources throughout ontogeny, complex life cycles also involve functional trade-offs and thereby create a new problem: how to optimize the transition between life stages [7,8].

The challenge of adapting to multiple adaptive peaks can be partially resolved by genetic and developmental decoupling among life stages [3]. Nonetheless, it is often the case that genetic, developmental, and functional correlations persist across life stages (e.g., [9-14]). Moreover, even if there is complete adaptive decoupling of divergent life stages, the transitional period between life stages is still likely to be a performance trough that exposes individuals to increased risks. Indeed, the more differentiated the life stages, the more intense the risks are likely to be. Metaphorically, the transition from juvenile to adult may be viewed as movement between alternative peaks on an individual's adaptive landscape [15,16], where peaks represent correspondence between an individual's phenotypic traits and the local maximum probability of survival.

Many amphibians exhibit a complex life cycle in which larval development (intervals of which are referred to as Gosner stages in frog tadpoles; [17]) is followed by metamorphosis into an adult form [5,18]. Tadpoles are highly specialized for feeding, and the tadpole body plan consists mostly of a globose body and a sheet-like, laterally compressed tail [19]. During metamorphosis, the tail is resorbed as hind and forelimbs emerge, thereby facilitating the transition from an aquatic swimming form (undulatory, axial locomotion) to a terrestrial hopping form (saltatory, appendicular locomotion). It is during the intermediate stages of metamorphosis that individuals are thought to experience increased predation risk [7]. The hypothesized period of increased predation risk separating larval and adult forms derives from the observation that metamorphs are optimized for neither larval nor adult niches [20]. For example, emergent hind limbs may impose drag and reduce swimming performance [19-21] and residual tail tissue may negatively impact saltatory locomotion [7]. For instance, metamorphosing chorus frogs, *Pseudacris triseriata*, are more likely to be captured by predatory garter snakes, *Thamnophus sirtalis*, than are either tadpoles or adult frogs [7]. Laboratory selection experiments on tadpoles likewise suggest the presence of a performance decline at metamorphosis [22]. Field experiments designed to measure both natural selection and variation in viability during ontogenetic stages near the developmental switch between life-stages, however, are still lacking.

Here, we test a set of related hypotheses about variation in survival probability in the wood frog, *Rana sylvatica*. We begin broadly, by first testing whether fitness (i.e., survival) correlates with morphology across tadpole development [22-25]. This first question is designed to test the hypothesis of increased predation risk during tadpole metamorphosis. We next use path analytic models to compare alternative hypotheses regarding the causal structure underlying selection on tadpole morphology. This includes a test of the hypothesis that tail morphology is subject to selection via its effect on swimming performance [24,26], which may be important for predator escape. We also consider the alternative hypothesis that tail shape may, as has been demonstrated previously, enhance survival by serving as a "lure" to attract predatory attacks towards the tail, thereby reducing the probability of mortal wounds to the head/body region [25,27-30].

## Methods

We collected tadpoles of the wood frog, *Rana sylvatica*, from a single pond near Randolph, Vermont, USA (43°54' N, 72°38' W) on June 11, 2010. The pool naturally contained larval odonates and other predatory invertebrates (Calsbeek and Kuchta, pers. obs.). Tadpoles were held overnight in 5 gallon food-grade plastic buckets with filtered pond water, and were fed an *ad libitum *diet of boiled lettuce. The morning after capture, we individually marked each tadpole with a unique color-coded combination of elastomer dyes (visible elastomer implants available from Northwest Marine Technologies, Shaw Island WA, U.S.A.) that we injected into the dorsal half of the tail fin, posterior to the body wall. Tadpoles were immobilized (but not anesthetized) during the marking procedure by holding them in a plastic multi-channel pipette well. We scored each tadpole's developmental stage [17] with the aid of a dissecting microscope just prior to the initiation of the selection experiment (mean Gosner stage = 34 ± 4.62 SD).

Tadpoles were then individually transferred to a V-shaped glass tank (which imposed a consistent orientation on the tadpoles) with a size standard, and were digitally photographed. We used digital images of each tadpole to make the following linear measurements: Head length: the distance from the anterior tip of the snout to the junction of the body with the tail; head height: the depth of head at its tallest point; tail length: from the junction of the tail with the body wall to the distal tip of the tail; tail muscle height: muscle height at the tallest point of the tail muscle; and tail fin height: fin height at the tallest part of the tail.

We measured swimming performance for half of the individuals in our selection study (N = 200 tadpoles) using a small (36L × 26W × 5H cm) tank containing filtered pond water and a size standard. Rapid development among the tadpoles held in buckets prevented us from measuring swimming performance for the remaining 200 individuals. Swimming trials were videotaped at 250 frames/sec using a high-definition digital camcorder (JVC Evario GZ-HM550-bu). Each tadpole was introduced to the swimming chamber and then motivated to initiate a "C-start" by touching the junction point between the tail fin and the body wall using a small metal pointer. We recorded three C-starts for each tadpole and used the fastest of these trials to estimate swimming performance, recording average speed over the 50 fastest frames. We chose to use this measure in our selection analyses because fifty frames was the average time required to swim one body length, and we assume that this is a good metric for predator avoidance. Burst speed was measured along the path of the tadpole movement using MotionAnalysis software (available from M. Chappell, University of California, Riverside, CA, U.S.A. http://warthog.ucr.edu/). We used the tadpole eye as a landmark for tracking individuals. All capturing, marking, photography, and swimming performance trials were conducted within 36 hours and the tadpoles were immediately transferred to cattle tanks for the selection experiment.

### Selection experiment

We conducted our selection experiment using eight 1136 L (300 gallon) cattle tanks that were randomly selected from an array of 49 tanks housed in an open field near the Dartmouth College campus. One month before introducing tadpoles, cattle tanks were cleaned and filled with ground water, 0.550 kg of dried Oak leaf litter, 15.4 g of rabbit chow (a nutrient source), and a three-liter aliquot of mixed zooplankton and phytoplankton collected from a pond near Norwich, VT (43.73° N, 72.31°W). We added five larval dragonflies (*Anax junius*) to each tank to serve as predators on tadpoles. To provide developing frogs with retreat sites, we placed three White Water Lilly (*Nymphea ordorata*) fronds on the water surface of each tank. To provide dragonfly larvae with perches, we used stones to anchor three to five tree branches (~100 cm) to the bottom of each tank. Finally, we randomly assigned 50 tadpoles to each of the eight tanks. Set up this way, the cattle tanks functioned as self-sustaining mesocosms that mimicked conditions experienced by tadpoles in nature [31]. We covered each cattle tank with 0.5 × 0.5 cm hardware cloth pulled taut and secured with elastic cords. This functioned to shade the tanks, prevent predation, ensure that no metamorphosing individuals escaped, and preclude large, predatory insects from laying eggs.

We recorded the identity of individual surviving tadpoles in our selection experiment five and fourteen days following introduction to the artificial ponds. Survival was scored by removing all the leaf litter and filtering each tank to recover tadpoles with hand-held dip nets. We also verified the presence of all five dragonfly-larvae in each mesocosm (one dragonfly-larva in each of three tanks was replaced to account for single dead individuals). After the first census, we replaced the leaf litter, dragonfly-larvae, and tadpoles, and re-covered the tanks with the shade cloth. Following the second census, all tadpoles were brought back to the laboratory, sacrificed with an overdose of MS-222, and stored in 70% ethanol.

General linear models were used to calculate selection gradients [32,33] for linear (β) and non-linear (γ) forms of selection. First, competing models were compared using Akaike's information criterion (AIC, [34] Table 1). This metric, which does not require nested models, calculates the likelihood of the model given the data and the number of parameters. Consequently, two models with equal likelihoods that differ in the number of parameters will have different AIC values, and the model with the smaller number of parameters will be favored. Next, the difference between the preferred model (i.e., with the lowest AIC value) and each of the subsequent models was calculated (Δ*i*) by stepwise inclusion of the remaining linear and quadratic terms. We then calculated the normalized relative likelihoods of the models, also known as the Akaike weights (*w_i_*), which quantify the relative support for different models [35]. Finally, we calculated the evidence ratio, which compares each model to the best model and provides the relative odds of competing models.

**Table 1:** Comparison of alternative selection models showing the number of parameters (k) in each model.

Traits in the model	k	AIC	**Δ**_**i**_	Likelihoods	*w_i_*	Evidence Ratio
Preferred model	7	893.91	0	1	0.6066	
Head Height	8	895.7	1.79	0.4099	0.2478	2.4473
Tail Muscle Height	9	897.69	3.78	0.151	0.0916	6.6194
Head Height^2^	10	899.81	5.9	0.0523	0.0317	19.106
Tail Muscle Height^2^	11	901.23	7.32	0.0257	0.0156	38.8613
Tail Fin Height^2^	12	902.95	9.04	0.0108	0.0066	91.8356

AIC score = Akaike Information Criterion; Δ*_I _= *difference in AIC scores between the best model and subsequent model; ***w_i _***= normalized relative likelihood of the model given the data; Evidence Ratio = the relative odds that a model is the best given the data. The first row of the table represents the preferred model, which includes linear terms for Head Length, Tail Length, and Tailfin Height, and quadratic terms for Head Length and Tail Length (see Table 4). Subsequent rows show the influence of adding each indicated trait in succession. Traits labeled with a superscript "2" represent quadratic terms.

Linear selection gradients were calculated from models that included only linear terms, whereas quadratic gradients (e.g., non-linear selection) and cross-product terms (i.e., correlational selection) were calculated from models that included both the linear and quadratic terms. Because the GLM underestimates quadratic terms by half [36,37], quadratic gradients and their standard errors were doubled. Though parametric statistics provide robust estimates of selection gradients and other parameters [32,38], these tests may be violated by survival data (live/die), which tend to have non-normally distributed errors [39,40]. We computed significance values for selection gradients using generalized linear models including a logit link function [41]. Prior to pooling data from individual tanks (i.e., replicates), we tested for any interaction between relevant terms and the factor for tank. None of these were significant, indicating that selection operated in the same way in all replicates. We dropped the interaction terms but retained a factor for "tank" in our models. The factor for tank explained a significant portion of the variance in all full models (0.02 > P < 0.03), but not in reduced models (0.06 > P < 0.08). All variables used in selection analyses were standardized to a mean of zero with unit standard deviation, except our fitness variable (survival), which was scaled by the mean [32,42]. The degree of multi-colinearity among traits was assessed by estimating variance inflation factors (VIF; [43]), all of which were less than five. We visualized fitness surfaces using cubic splines [44].

**Figure 1:**
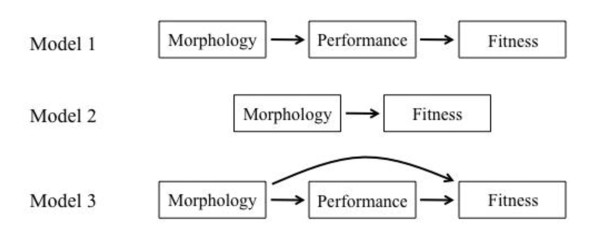
**Path diagram of the relationships between morphology, swimming speed, and fitness**. Model 1 is a classic path analysis diagram with links going from morphology to performance to fitness [62]. In Model 2, links go directly from morphology to fitness; performance is omitted. This would be the case if morphology impacted fitness through means other than swimming speed, for example by acting as a caudal lure. Model 3 is the "full model" and includes links between morphology and performance as well as between morphology and fitness, as would be the case if swimming speed as well as other factors mediated the relationship between morphology and fitness.

Path analysis was used to investigate the structure of causal relationships in our selection experiments [45,46]. First, we developed a set of *a priori *causal path models based on the competing hypotheses that variation in survival was most highly dependent on swimming performance versus predator evasion by caudal luring (Figure 1). The swimming performance model included causal paths from morphology → performance → fitness, while the caudal luring model was reduced to causal paths from morphology → fitness only. This latter case models a situation in which a trait other than burst swim speed mediates the relationship between morphology and fitness [47]. A third model combined the two models above, and allowed for the possibility that morphology impacts fitness through both measured and unmeasured performance variables. Significance tests for individual path models were based on comparisons in which the covariance structure of each model was tested against the covariance expected under the assumption the model was correct [48]. A significant difference in this comparison indicates that the model in question provides a poor fit to the data. Path analyses, including significance tests, were performed using the program AMOS v. 18 [49]

In a second analysis, our Model 3 (Figure 1) was iteratively reduced to its significant components by sequentially setting causal paths with the lowest partial regression coefficients and the highest *P *values to zero [50]. Alternative models were compared using Akaike's information criteria (AIC, [34]), including the difference between the preferred model and each subsequent model (Δ*i*), normalized relative likelihoods (*w_i_*), and evidence ratios [35,51].

In addition to path analysis using maximum likelihood, we also conducted Bayesian analyses of the data. We did this to account for the binomial distribution of our fitness variable (survival), which likely violates the assumption of normal errors and multivariate normality in least-square calculations [52,53]. Bayesian analysis in Amos 18 [49] employs a Markov Chain Monte Carlo (MCMC) algorithm for estimating posterior distributions, and properly accounts for the binomial status of our fitness variable. Parameter estimates were estimated from 150,000 generations following a burn-in of 500 generations. Convergence of the MCMC algorithm was assessed using the convergence statistic developed by Gelman et al. [54] and implemented in AMOS [49]. The significance of parameter estimates was assessed using 95% Bayesian credibility intervals. The results of the Bayesian analysis of the path coefficients were very similar to the maximum likelihood estimates (data not shown) and will not be presented.

## Results

Despite the presence of floating refugia in each tank, our artificial ponds turned up one metamorphosed frog that clearly drowned after failing to find a terrestrial refuge. It is likely that some fraction of the mortality that we attributed to predation was from metamorphs that drowned. On the other hand, we also recovered six fully metamorphosed frogs that survived to the end of the experiment. As a conservative approach to analyses, we broke our data up into two different data sets. The first dataset is based on all individuals in the study, and is referred to as the "Full" dataset; it is described throughout the rest of this paper. The second dataset excluded all individuals whose Gosner stage was > 39 at the experiment's outset; this is referred to as the "reduced" data set. Based on rates of development measured in our study, this reduced dataset increases the chance that most individuals were in an aquatic stage throughout much of the course of the experiment. Morphological measurements could not be made for a few individuals and the size of these data sets varies slightly (see table legends for details). Results from the two sets of analyses were qualitatively nearly identical (Tables 2, 3 and 4).

**Table 2:** Linear (β) and quadratic (γ) selection on all morphological traits measured in this study in a data set that included all individuals (Full, N = 172), as well as in a second dataset from which we excluded all individuals whose Gosner stage was > 39 at the time of release were excluded (Reduced, N = 151).

		Directional selection	Quadratic selection
		
Data set	Trait	β ± SE	γ ± SE
Full	Head Length	0.08 ± 0.16	- 0.06 ± 0.16
	Head Height	0.10 ± 0.12	0.14 ± 0.14
	Tail Length	- 0.16 ± 0.13	0.004 ± 0.16
	Tail muscle Height	- 0.14 ± 0.15	- 0.10 ± 0.14
	Tail Fin Height	0.22 ± 0.09*	- 0.06 ± 0.11
	Swimming speed	- 0.01 ± 0.07	- 0.06 ± 0.10
			
Reduced	Head Length	0.05 ± 0.19	- 0.10 ± 0.17
	Head Height	0.09 ± 0.13	0.14 ± 0.15
	Tail Length	- 0.17 ± 0.14	0.04 ± 0.19
	Tail muscle Height	- 0.08 ± 0.16	- 0.03 ± 0.17
	Tail Fin Height	0.21 ± 0.10*	- 0.05 ± 0.12
	Swimming speed	- 0.05 ± 0.08	- 0.10 ± 0.11

Asterisks indicate significant (* *P *< 0.05) selection, as determined by a generalized linear model with a logit link function and survival (0 or 1) as the response variable. A term for Gosner stage was included in these models, but because development stage is not a "trait" on which one can measure selection, is not shown here. A factor for tank was also included. See text for details.

**Table 3:** Linear (β) and quadratic (γ) selection on all morphological traits except burst swimming speed.

		Directional selection	Quadratic selection
		
Data set	Trait	β ± SE	γ ± SE
Full	Head Length	- 0.01 ± 0.11	- 0.18 ± 0.10
	Head Height	0.10 ± 0.09	0.04 ± 0.09
	Tail Length	- 0.19 ± 0.07*	- 0.16 ± 0.06*
	Tail muscle Height	0.02 ± 0.09	- 0.03 ± 0.09
	Tail Fin Height	0.24 ± 0.06***	- 0.07 ± 0.06
			
Reduced	Head Length	- 0.08 ± 0.12	- 0.24 ± 0.11*
	Head Height	0.07 ± 0.10	0.07 ± 0.10
	Tail Length	- 0.14 ± 0.08	- 0.11 ± 0.08
	Tail muscle Height	0.07 ± 0.10	-0.03 ± 0.10
	Tail Fin Height	0.16 ± 0.07*	-0.08 ± 0.08

The results of two analyses are presented: a Full dataset that included all individuals (N = 381), as well as in a second dataset in data set (Reduced, N = 324), from which we excluded all individuals whose Gosner stage was > 39 at the time of release. Asterisks indicate significant (* *P *< 0.05, ***P < 0.005) selection, as determined by a generalized linear model with a logit link function and survival (0 or 1) as the response variable. A term for Gosner stage was included in these models, but because development stage is not a "trait" on which one can measure selection, is not shown here. A factor for tank was also included. See text for details.

**Table 4:** Linear (β) and quadratic (γ) selection on morphological traits used in a model chosen based on AIC scores.

		Directional selection	Quadratic selection
		
Data set	Trait	β ± SE	γ ± SE
Full	Head Length	0.05 ± 0.09	- 0.20 ± 0.07*
	Tail Length	- 0.17 ± 0.07*	- 0.16 ± 0.06*
	Tail Fin Height	0.27 ± 0.05***	
			
Reduced	Head Length	- 0.007 ± 0.10	- 0.20 ± 0.08*
	Tail Length	- 0.11 ± 0.08	- 0.10 ± 0.08
	Tail Fin Height	0.20 ± 0.07***	

Two identical analyses are presented: the first included all individuals (Full, N = 381), the second is from a censured dataset (Reduced, N = 325) from which all individuals whose Gosner stage was > 39 at the time of release were excluded. Asterisks indicate significant (* *P *< 0.05, ***P < 0.005) selection, as determined by a generalized linear model with a logit link function and survival (0 or 1) as the response variable. A term for Gosner stage was included in these models, but because development stage is not a "trait" on which one can measure selection, is not shown here. A factor for tank was also included. See text for details.

In the full data set, the mean percentage survival (± SE) in each tank to the first census period (5 days) was 0.77 ± 0.02 (range 0.66-0.90). By the second census (14 days), mean survival had decreased to 0.57 ± 0.02 (range 0.48-0.66). Qualitatively, selection results during the two time periods were nearly identical (data not shown), but to maximize our power to detect selection, and to simplify the presentation of results, we use viability estimates from the second census as our measure of fitness. Frequent bite marks on the tails of surviving tadpoles suggest that dragonfly larvae were a key source of mortality in our study populations. We also recovered two complete tails during our census, with elastomer tags still intact, from tadpoles that did not survive. We conclude that mortality in the selection replicates was largely due to predation by dragonfly larvae.

**Figure 2:**
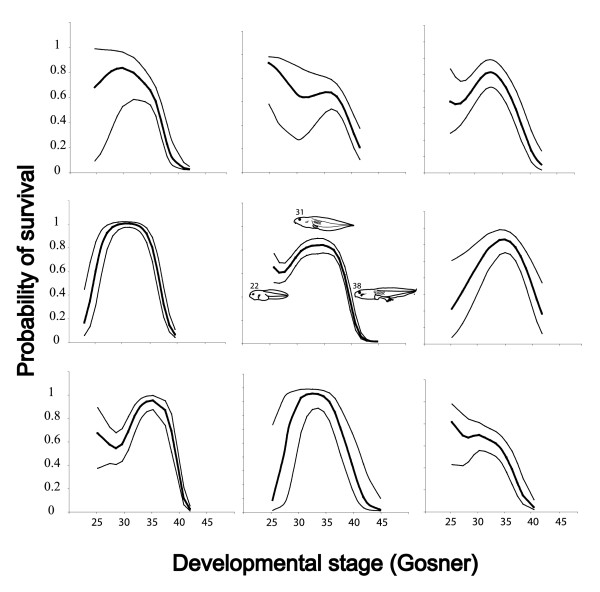
**The probability of survival was maximized for tadpoles in intermediate stages (e.g., Gosner stages 30-37) of development**. Variation in survival is shown for the eight selection replicates used in our experiment (outer panels) and for all replicates pooled (central panel). The dark line represents the best fit cubic spline and light lines indicate the 95% confidence limits. The central panel includes illustrations of representative tadpoles/metamorphs at several developmental stages (indicated as numeric Gosner stages above each illustration).

**Figure 3:**
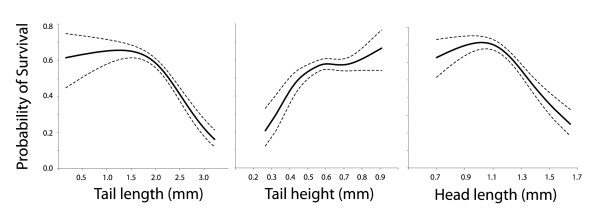
**In the preferred model (Table 3) chosen based on AIC scores (Table 1), natural selection acted primarily on tail length, tail height and head**. Selection on tail length and head length both were stabilizing around shorter values. Selection on tail height was directional and positive. See the text and Table 3 for statistical details. The dark line represents the best fit cubic spline for each trait, and light lines indicate the 95% confidence limits.

Variation in survival was strongly linked to ontogeny (Gosner stage) and favored tadpoles at intermediate stages of development (quadratic effect of Gosner stage ANOVA F_1,385 _= 63.83, P < 0001) with a decline in survival probability starting, on average, around Gosner stage 37 (Figure 2, center panel). We therefore included a term for Gosner stage in selection models. For completeness, we present models that include all measured traits (Table 2), models without swimming speed (which maximizes our sample size; Table 3), and a model using the set of independent variables corresponding to the smallest AIC score (Table 4). This last model, which we consider the preferred model (Table 1), included a linear term for tail fin height and Gosner stage, and linear and quadratic terms for tail length and head length. In this model, selection favored individuals with deep tail fins (β = 0.27 ± 0.05, P < 0.0001) and short tails (β = -0.17 ± 0.07, P = 0.02) (Figure 3). We also detected quadratic components to selection on tail length and head length that were both stabilizing (tail length: γ_1,1_, = -0.16 ± 0.06, P = 0.01; head length: γ_2,2 _= -0.20 ± 0.07, P = 0.01) (Figure 3). To verify that the results were not biased by the relationship between size and Gosner stage, we regressed Gosner stage against tail and head morphology, and saved the residuals. Patterns of selection based on residual trait values were qualitatively similar to those using raw values (e.g., selection for deep residual tail fins [β = 0.22 ± 0.04, P < 0.0001] and short residual tail lengths [β = -0.10 ± 0.04, P = 0.03]).

Swimming speed was positively correlated with developmental stage (r^2 ^= 0.14, df = 186, P < 0.0001) and tail length (r^2 ^= 0.12; df = 186, P < 0.0001), and there was a weak quadratic relationship between swimming speed and tail fin height (individuals of intermediate tail fin height swam fastest: r^2 ^= 0.07, df = 186, P = 0.051). However, we did not detect any selection on swimming speed in our experiment. In a model that included linear terms for tail length and tail fin height, the selection gradient for swimming speed was weakly negative and non-significant (β = -0.03 ± 0.07, P = 0.65). Even when we considered selection on swimming speed alone (i.e., the selection differential for swimming speed) we detected no variation in survival that was related to swimming performance (s = -0.07 ± 0.06, P = 0.23).

The results of the path analyses parallel the multiple regression analyses. The best fit model was Model 2 (Morphology → Fitness; AIC = 52.41; DIC = 1056.30; Figure 1; Table 5). Over 99% of the relative likelihood was captured by this model, and the relative odds of the second best model being better than the most strongly supported model was 3159:1 (Table 5). In contrast, Model 1 (Morphology → Performance → Fitness; Figure 1) was significantly different from the data (χ^2 ^= 46.88; *P *< 0.001). We thus conclude that Model 2 is strongly supported relative to alternatives.

**Table 5:** Comparison of path models.

	Maximum Likelihood Analyses						
Model	**χ**^**2**^	*df*	*P*	AIC	**Δ**_***i***_	*w_i_*	Evidence Ratio
Morphology -- > Fitness	0.41	1	0.52	52.41	0	0.9997	
Full Model	0.53	1	0.47	68.53	16.12	3.16 × 10^-4^	3158.97
Morphology -- > Performance -- > Fitness	46.88	5	< 0.001	106.88	54.47	1.48 × 10^-12^	6.74 × 10^11^

Chi-square values (χ^2^), degrees of freedom (*df*), and the associated *P *value report the significance of the model. AIC = Akaike Information Criterion; Δ*_I _= *difference in AIC scores between the best model and subsequent model; ***w_i _***= normalized relative likelihood of the model given the data; Evidence Ratio = the relative odds that a model is the best given the data.

**Figure 4:**
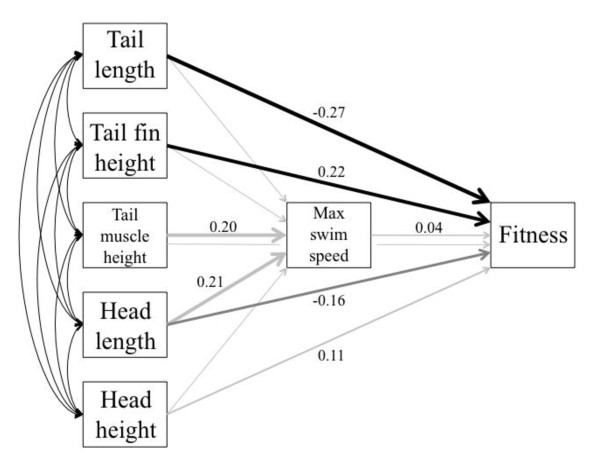
**Path diagram of the relationships between morphology, swimming speed, and fitness**. The results of model 5 (Table 5) are illustrated here. Double-headed arrows represent covariances (range: 0.551 - 0.854), and all of them are significant. Values near single-headed arrows are maximum likelihood parameter estimates of partial regression coefficients (direct effects. Arrows lacking a number represent causal paths set to zero (Table 5). Arrow thickness is proportional to the strength of relationship. Black arrows present significant parameter estimates, and grey arrows represent relationships that are not significant. The *P*-value of the dark grey arrow (head length → fitness) is 0.06. Note that the causal paths between head length → fitness and head length → maximum swim speed are significant in model 9, which had the best AIC score (Table 6).

In our second path analytic approach, we iteratively reduced causal paths by removing the most poorly supported paths after each run until we were left with a model in which all causal paths were significant. We started with the Full Model (Model 3; Figure 1) because this model included all theoretically interesting causal paths. The most fully reduced model received the strongest support (AIC = 61.38; Table 6). In this best fit model, the only significant causal paths were between tail fin height and fitness (β = 0.24; *P *< 0.001) and tail length and fitness (β = -0.32; *P *< 0.001). However, the best-fit model was not a robust improvement over related models. For example, the difference in AIC between the best-fit model and the 4^th ^best model was only 1.00, and for the 5^th ^best model, 2.19 (Table 6). In addition, the relative odds of models 2-4 ranged from 1.35-1.65:1, and the relative odds of model 5 was 3:1 (Table 6). We conclude that the first five models are not easily distinguished, we therefore show the results of Model 5 in our path analysis diagram because it is the fullest model receiving statistical support (Figure 4). Relative to the best-fit model, the 5^th ^best model includes causal paths between head height → fitness, head length → fitness, tail muscle height → maximum swim speed, and maximum swim speed → fitness (Figure 4). None of these were significant, however, there is a trend between head length → fitness (β = -0.16; *P = *0.06). Note that the link between swimming performance → fitness is weak and not significant (β = 0.04; *P *> 0.05).

**Table 6:** Comparison of path models.

		Maximum Likelihood Analyses						
Model	Path constrained to zero	**χ**^**2**^	*df*	*P*	AIC	**Δ**_***i***_	*w_i_*	Evidence Ratio
1	Head length → Fitness	5.38	7	0.61	61.38	0	0.28	
2	Max. swim speed → Fitness	1.98	5	0.85	61.98	0.60	0.21	1.35
3	Head ht. → Fitness	4.28	6	0.64	62.28	0.89	0.18	1.56
4	Tail muscle ht. → Max. swim speed	8.39	8	0.40	62.39	1.00	0.17	1.65
5	Tail fin ht. → Max. swim speed	1.58	4	0.81	63.58	2.19	0.09	3.00
6	Tail length → Max. swim speed	1.20	3	0.75	65.20	3.81	0.04	6.73
7	Tail muscle ht. → Fitness	0.89	2	0.64	66.89	5.51	0.02	15.70
8	Head ht. → Max. swim speed	0.36	1	0.55	68.36	6.98	0.01	32.77
9	Saturated model	--	0	--	70.00	8.62	0.00	74.33

Chi-square values (χ^2^), degrees of freedom (*df*), and the associated *P *value report the significance of the model. Note that model 9 is saturated, and thus the fit of the model to the data could not be tested using the chi-square statistic. In models that are not significant, the data are a good fit to the model. AIC = Akaike Information Criterion; Δ*_I _= *difference in AIC scores between the best model and subsequent model; ***w_i _***= normalized relative likelihood of the model given the data; Evidence Ratio = the relative odds that a model is the best given the data.

## Discussion

One of the most common hypotheses regarding the evolution of complex life cycles is that alternative morphological strategies are employed to exploit different resources throughout ontogeny. The transition between life stages, however, can be a vulnerable period in which individuals suffer higher rates of mortality. We have presented empirical evidence that tadpoles of the wood frog, *Rana sylvatica*, when facing predation by dragonfly larvae, experience a higher probability of mortality as they approach metamorphic climax. That mortality probabilities increase during metamorphosis is not unexpected, as a tadpole with emergent hind and forelimbs is well adapted for neither swimming nor jumping [7,20,21]. For instance, Arnold and Wassersug [8] showed across a large geographic range (Mexico to Washington state) that garter snakes, *Thamnophis spp*., were more likely to have consumed anuran metamorphs (tree frogs and toads) than either tadpoles or adults. They concluded that transforming anurans were highly susceptible to snake predation as a consequence of "locomotor ineptitude."

Our data further suggest that selection acts strongly on morphological traits, favoring tadpoles with short tails and deep tail fins, but that this selection acts largely independently of swimming performance. This latter result is surprising given that tail shape influences swimming performance [55]. Indeed, in our data swimming performance was correlated with both tail length and tail fin depth, and larger values of both tail elements produced greater swimming speeds, consistent with patterns demonstrated elsewhere [56,57]. Our analyses may have suffered from reduced power given that we could only measure swimming speed for half of our study animals. However, even when we removed all other terms from the model and measured selection differentials on swimming speed alone, the results were not significant. Moreover, path analyses revealed that the effects of morphology (tail length and tail fin height) were largely direct, acting to enhance survival probability per se, rather than serving as a functional link to swimming performance. We interpret this result as consistent with the hypothesis that short tails and deep tail fins are adaptive because they attract predatory strikes and increase the probability that a predator will strike tail tissue rather than sites on the head or body (i.e., "the caudal lure hypothesis"; [25,27,30]).

Tadpoles of many frog species exhibit developmental plasticity in response to chemical cues from potential predators, whereby they develop a relatively deep tail fin and a small body (*e.g*., [22,58-61]). In particular, enlarged tail fins lead to enhanced survival in the presence of larval odonates (summarized in [58]). There is reason to believe, however, that differences in tail shape do not influence swimming performance effectively enough to have a large impact on survival in the presence of odonate larvae. This result is unexpected at first blush, given the high prevalence of causal relationships between morphology and performance in other animal systems [62-64]. Van Buskirk and McCollum [24] used experimental manipulation of tail fin morphology, trimming tissue to reduce both the total length and depth of the tail fin, to investigate the direct effects of changes in tail morphology on swimming performance. Their study revealed that changes in swimming performance were not apparent until one third of the tail was surgically removed, leading them to conclude that reduced susceptibility to predation must have been due to something other than enhanced swimming performance. Similarly, Wilbur and Semlitch [65] showed that damaged tails of *R. utricularia *incurred little survivorship cost in the presence of predatory newts (*Notophthalmus viridescens*). On the other hand, Van Buskirk et al [28] showed that tadpoles with predator-induced morphologies suffered fewer lethal strikes to the body, suggesting that enlarged tail fins may enhance survival via a "caudal lure" effect.

The approach adopted in this study was to quantify relative survival and selection across ontogeny. One challenge faced by such an approach is that changes in size and shape are confounded throughout the development of the tadpole. This is the phenomenon summarized by Gosner stages. In addition, we were only able to quantify swimming performance and morphometric variables at the start of the study. Depredated tadpoles, unfortunately, cannot be measured. Our analyses thus assume that fundamental elements of size and shape were captured in our initial measures, and that the quantitative signal is maintained to some degree throughout ontogeny. If this were not the case, it is unlikely that we would have obtained sensible results.

Though the number of studies of natural selection has grown rapidly in recent decades [66,67], there are still fundamental gaps in our understanding of the selective process. This is, in part, owing to the fact that selection studies are rarely replicated either temporally or spatially [41] and when studies are replicated, selection estimates tend to be highly variable among replicates [68]. Our study provides a rare example of repeatable selection, as replicate estimates of selection were highly congruent among mesocosms, suggesting that the changes that characterize metamorphosis are subject to strong and consistent patterns of selection among individuals.

## Conclusions

Our study demonstrates an increase in mortality risk as tadpoles began to metamorphose. Owing to the nature of our experimental design, which focused on tadpole mortality, our data did not examine the effects of the transition from tadpole to froglet on survivorship (see [8]). As metamorphosis proceeds and the tail fin is resorbed, we expect that froglets would become better at hopping and thus less susceptible to predation. We suggest, as have others [19], that selection should thus favor individuals that minimize the transition time during metamorphic climax. This does not necessarily mean that selection should favor the most rapid possible development. Indeed, faster overall development often results in small adult body sizes, a condition that can have serious fitness consequences for adult anurans [69,70]. Rather, the optimal strategy should be to metamorphose at a rate that maximizes the balance between the probability of surviving metamorphosis and later fitness costs. Future studies should aim to measure selection on the separate components of developmental timing to improve our understanding of the targets of selection, including the costs and benefits of pursuing alternative metamorphic strategies.

## Authors' contributions

RC and SK designed and carried out the experiments, analyzed the data, and wrote the manuscript. Both authors read and approved the final manuscript.
